# Salmonellosis outbreak archive in China: data collection and assembly

**DOI:** 10.1038/s41597-024-03085-7

**Published:** 2024-02-27

**Authors:** Zining Wang, Chenghu Huang, Yuhao Liu, Jiaqi Chen, Rui Yin, Chenghao Jia, Xiamei Kang, Xiao Zhou, Sihao Liao, Xiuyan Jin, Mengyao Feng, Zhijie Jiang, Yan Song, Haiyang Zhou, Yicheng Yao, Lin Teng, Baikui Wang, Yan Li, Min Yue

**Affiliations:** 1 Department of Veterinary Medicine, Zhejiang University College of Animal Sciences, Hangzhou, 310058 China; 2 Hainan Institute of Zhejiang University, Sanya, 572000 China; 3 Zhejiang Provincial Key Laboratory of Preventive Veterinary Medicine, Hangzhou, 310058 China; 4 State Key Laboratory for Diagnosis and Treatment of Infectious Diseases, National Clinical Research Center for Infectious Diseases, National Medical Center for Infectious Diseases, The First Affiliated Hospital, College of Medicine, Zhejiang University, Hangzhou, 310003 China

**Keywords:** Bacterial infection, Developing world, Bacteriology, Risk factors

## Abstract

Infectious disease outbreaks transcend the medical and public health realms, triggering widespread panic and impeding socio-economic development. Considering that self-limiting diarrhoea of sporadic cases is usually underreported, the *Salmonella* outbreak (SO) study offers a unique opportunity for source tracing, spatiotemporal correlation, and outbreak prediction. To summarize the pattern of SO and estimate observational epidemiological indicators, 1,134 qualitative reports screened from 1949 to 2023 were included in the systematic review dataset, which contained a 506-study meta-analysis dataset. In addition to the dataset comprising over 50 columns with a total of 46,494 entries eligible for inclusion in systematic reviews or input into prediction models, we also provide initial literature collection datasets and datasets containing socio-economic and climate information for relevant regions. This study has a broad impact on advancing knowledge regarding epidemic trends and prevention priorities in diverse salmonellosis outbreaks and guiding rational policy-making or predictive modeling to mitigate the infringement upon the right to life imposed by significant epidemics.

## Background & Summary

Outbreaks, being intricate public health events, not only entail medical implications but also pose catastrophic consequences for public sentiment and socioeconomic development^1,2^. As the COVID-19 pandemic continues to make a global impact, the proactive measures undertaken by the Chinese government, particularly the implementation of non-pharmaceutical interventions and predictive modeling based on outbreak trends, have substantially mitigated the incidence rates (IR) of various infectious diseases^3–6^. This underscores the growing imperative to understand infectious disease trends and underlying factors in many countries, including China. Salmonellosis is a clinically symptomatic infection caused by various *Salmonella* serovars. This prevalent foodborne illness is mainly attributed to diverse serovars through consuming contaminated food or water^7–10^. In humans, the most common symptoms of classical nontyphoidal *Salmonella* (NTS) are diarrhea, fever, abdominal cramps, and vomiting^11–14^. Occasionally, specific populations with weakened immune systems likely lead to severe disease, including death outcomes. Specific serovars, particularly typhoidal *Salmonella*, can result in typhoidal fever (TF) or paratyphoid fever (PTF)^15^.

As the leading global cause of diarrheal disease, *Salmonella* infection leads to approximately 131 million cases, resulting in 370,000 deaths annually^16^. *Salmonella* is also among the most common agents, causing notorious local, national, and international outbreaks. Certain high-income countries like the United States^17^, the United Kingdom^18^, and France^19^ have conducted long-term studies on the spatiotemporal patterns and prevention priorities of *Salmonella* outbreaks (SO). Even though it is frequently pressed in various media, systematic investigations regarding SO, in developed countries like China, are largely lacking.

The Chinese CDC estimates *Salmonella* is the cause of 70–80% of bacterial foodborne illnesses, ranking as one of the top two diarrheal agents^20^. A substantial body of research and literature on SO in China has been documented, which parallels the CDC estimations. However, qualified datasets and knowledge gaps in SO remain significant rational policy-making barriers. Specifically, the scope of available studies is limited, as they fail to comprehensively represent the overall situation, including various serovars^21^, different regions^22^, multiple antimicrobial resistance^23^, and the diversity of animal-derived food sources^24^. Previous studies often focused on individual outbreak cases, employing epidemiological methods such as case-control studies and various biological tracing approaches^25^. While these approaches provide valuable insights, they have significant limitations when applied to large-scale data analytics and predictive modeling^26^. Here, we conducted a systematic review, meta-analysis, and correlation analysis of the extensive studies to uncover the epidemiological patterns of SO in China, which lay the baseline for future policy-making and ultimate predictive modeling of outbreaks of salmonellosis.

**Fig. 1:**
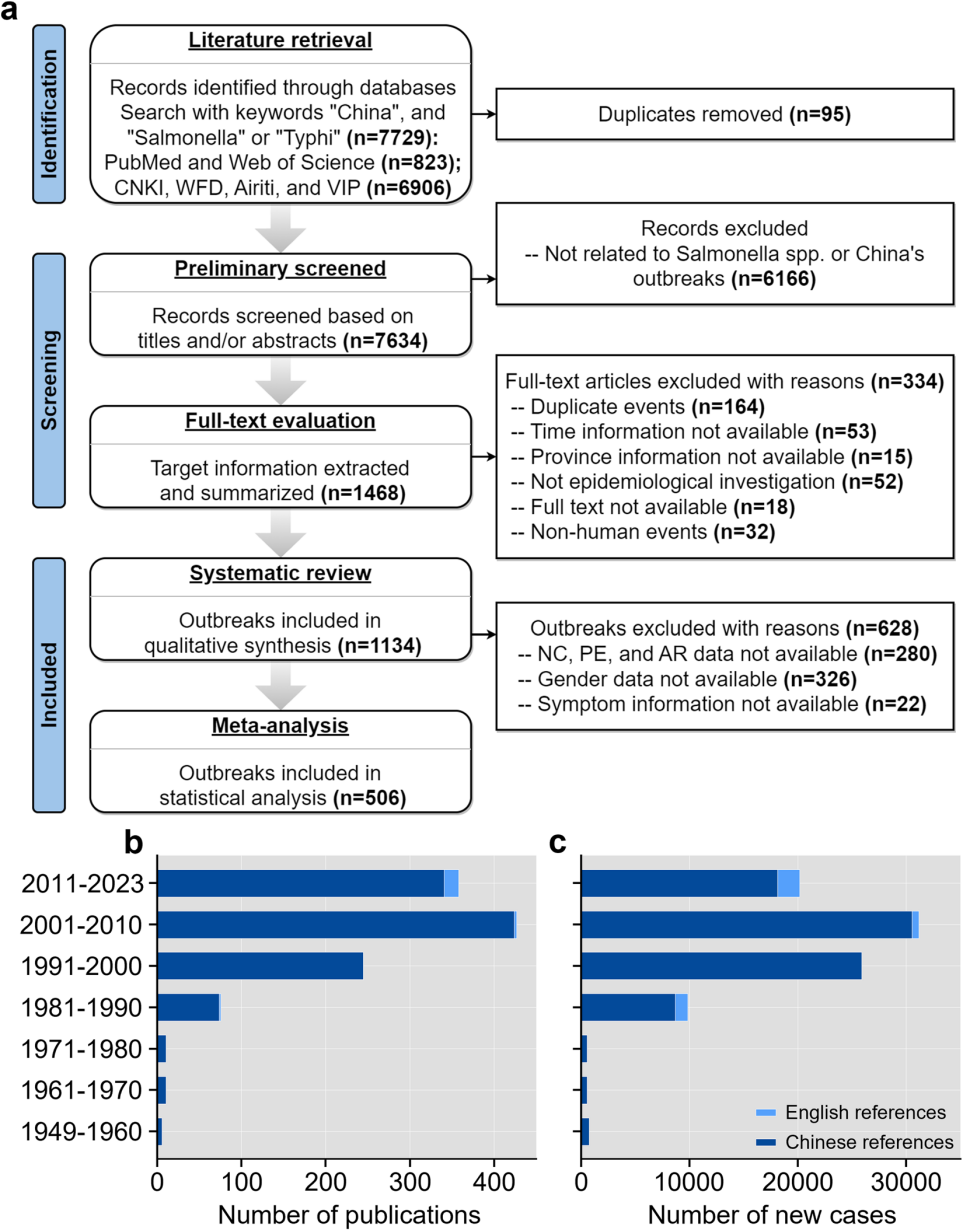
Analysis process and dataset characteristics of systematic review and meta-analysis. (**a**) Preferred Reporting Items for Systematic Reviews and Meta-Analyses (PRISMA) flow diagram of search strategy and selection of articles. (**b**) The number of Chinese and English publications included in the systematic review dataset by year period. (**c**) The number of new cases recorded in Chinese and English publications included in the systematic review dataset by year period.

A total of 1,134 outbreaks involving over 89,050 patients were included in this systematic review, with 506 high-quality reports incorporated into the meta-analysis (Fig. 1). This study has limitations, primarily related to the potential reporting bias in outbreak recording in the current system. 1) Factors such as economic, educational, and healthcare levels can influence the attention given by researchers to SO and the timeliness of reporting. 2) Relying solely on literature collection as the studied dataset was the current trade-off approach, the Chinese Pathogen Identification Net, led by the National Center for Disease Control and Prevention, would provide a more extensive dataset for understanding the salmonellosis burden in China. There is an urgent need for open access and data transparency from the Chinese surveillance and reporting system^27^. Nonetheless, this study illuminates underappreciated knowledge of the epidemiological trend due to SO in China, serving as a baseline for understanding the burden of salmonellosis and informing further outbreak prediction.

## Methods

Following the PRISMA statement and the STROBE statement^28,29^, a systematic review with a meta-analysis approach registered on the PROSPERO for observational studies was conducted to investigate the epidemiological patterns and prevention priorities of SO in China from 1949 to 2023 (CRD42023458488).

### Search strategy

To guarantee the comprehensiveness of the research materials, an exhaustive search was conducted using six databases in both English and Chinese. The English databases consisted of Web of Science (WOS) and PubMed, while the Chinese databases incorporated China National Knowledge Infrastructure (CNKI), Wanfang Data (WFD), Airiti Library, and China Science and Technology Journal Database (VIP). Due to the variety of terminology used to describe outbreaks, the search strategy entailed the utilization of the keywords “*Salmonella*” or “Typhi” in combination with “China” to identify all relevant published literature potentially associated with SO in China before March 31, 2023. Non-Chinese and non-English literature were not included in this study.

After the online systematic evaluation tool Rayyan and manual proofreading, 95 duplicates were identified and removed^30^. 7,729 retrieved and de-duplicated pieces of literature were included in the subsequent eligibility criteria screening, comprising 823 English and 6,906 Chinese published documents (available at Figshare).

### Inclusion and exclusion criteria

For the qualifying SO events, as our primary aim, a two-stage screening process was undertaken, consisting of literature screening and event screening. The list of literature included in the screening catalogue was independently assessed for eligibility based on titles and abstracts by different authors, and at least one reviewer considered the screening results of the previous author after a full-text review. Three authors conducted all subsequent systematic review processes in parallel, and any discrepancies were resolved through discussion.

The preliminary screened literature encompassed various types, including case reports, laboratory pathogen tracing analyses, and epidemiological investigations. Nevertheless, editorials, commentaries, conference presentations, or relevant retrospective reviews were excluded. In addition, literature not related to *Salmonella* or reports of outbreaks outside China were excluded. To perform an epidemiological analysis based on outbreak events, the full-text screening of 1,468 pre-screened articles excluded outbreaks without epidemiological investigation information or outbreaks for which full-text access was unavailable. Given the heterogeneity in literature structure, spatiotemporal characteristics were established as a primary criterion, and outbreaks without valid information on year, month, and province were excluded. Duplicate reports of the same outbreak were excluded by determining the independence of outbreaks based on spatiotemporal characteristics. Finally, after the exclusion of non-human SO, a total of 1,134 outbreaks were included for subsequent data extraction and systematic analysis.

### Data extraction and quality assessment

**Fig. 2:**
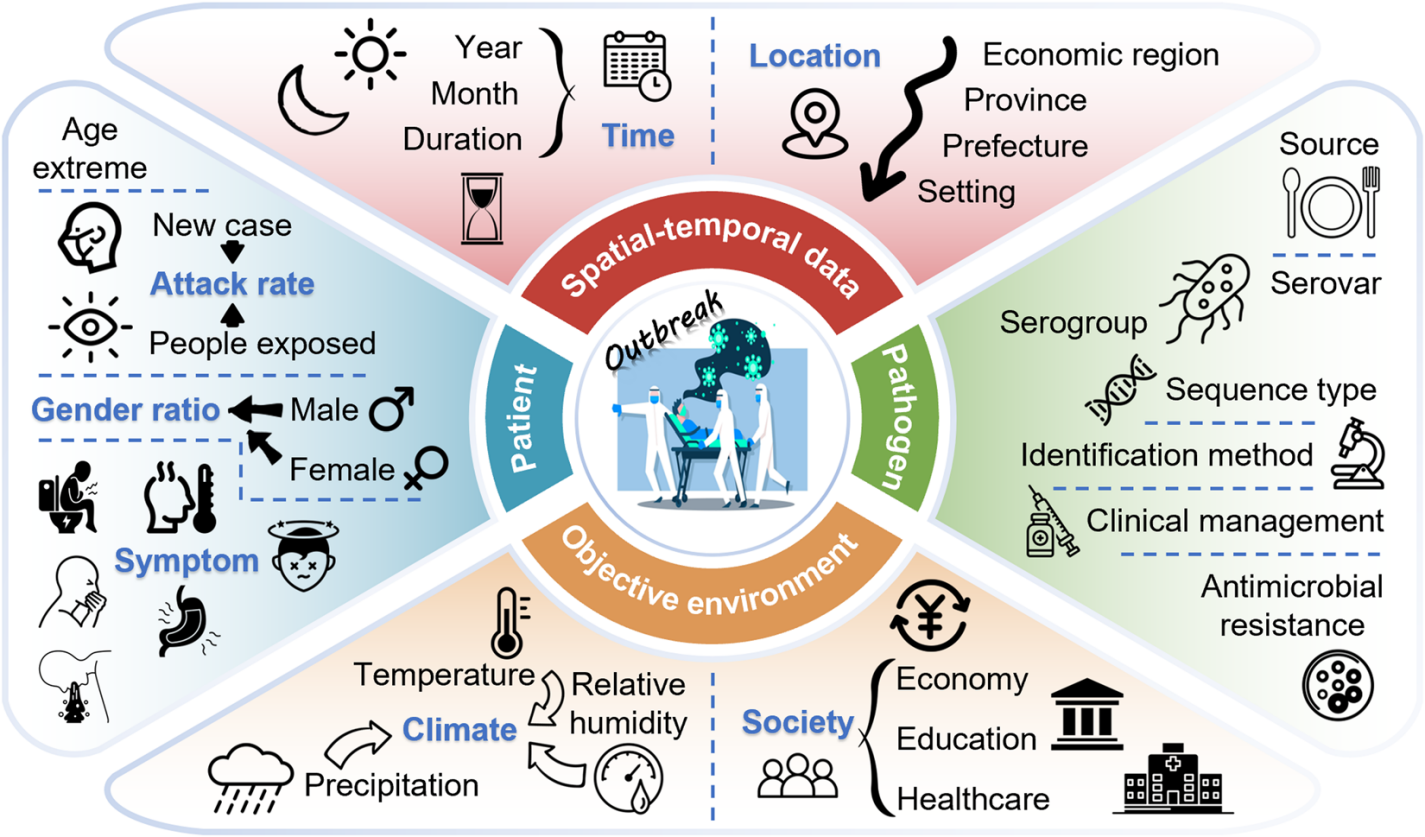
Dataset composition and structure. The complete outbreak datasets are divided into four parts: temporal-spatial data, patient cohort information, pathogen diagnosis and treatment, and objective environmental factors.

Research data acquisition involved two primary approaches: online database downloads and comprehensive full-text extraction (available at Figshare). Data from database downloads encompassed author names, article titles, journal names, volume, issue, page numbers, publication year, and digital object identifier (DOI). The comprehensive full-text extraction yielded data in four distinct categories: spatiotemporal background, patient information, laboratory tests, and clinical management. The category information included the following details: onset year and month, duration, province, prefecture, and setting of the outbreak; the number of new cases (NC) and people exposed (PE); minimum, maximum, and average age; the standard deviation of age; the number of male (M) and female (F) patients; IR of designated symptoms such as diarrhea, fever, stomach cramps, nausea, vomiting, and headache; food sources; serogroups, serovars, sequence types, antimicrobial resistance profiles, and identification methods; antimicrobial treatment regimens and prognosis. Additionally, the extracted information was utilized to calculate hysteresis bias (HB), attack rate (AR), IR, and gender ratio (GR). Furthermore, a substantial volume of public data has been collected for correlation analysis (Fig. 2).

The quality assessment and risk of bias of included outbreak studies were evaluated using the Joanna Briggs Institute checklist for reporting prevalence data, with appropriate modifications^31^. Five criteria were adopted in the assessment: event overview, study population, identification method, description of statistical analysis, and clinical information (available at Figshare). Each study was scored based on the checklist, with two score options (2 or 1) for each criterion. Only studies scoring high for quality and low for risk of bias (total score = 10) were included in the meta-analysis.

## Data Records

The data are available from the Figshare platform as XLSX files with multiple tabs upon reasonable request with a CC-BY license (10.6084/m9.figshare.24033690)^32^. Each row in the table corresponds to an outbreak record, and each column represents a variable whose default interpretation and data profile are shown below (N/A means no available data):

- Publication information: The author, title, journal, and DOI information for each publication are listed to facilitate data source tracking and verification by authors or subsequent dataset users. Information from all Chinese journals is separately translated into English (E.T.). The document serial numbers have no practical significance and serve only as a counting function for arbitrary sorting.
- Spatiotemporal background: The year and month serve as the outbreak’s initiation time, distinct from the publication date. The recorded duration is defined as the number of days between the date of the first confirmed case and the diagnosis date of the last case, with a minimum duration of one day. Economic regions and administrative regions are referenced from official documents of the National Bureau of Statistics of China. In cases of cross-border transmission, the outbreak’s initiation location is defined by the spatial information in the record. The latitude and longitude of the outbreak are calculated based on the central coordinates of the prefectural-level city. Specific occurrence settings are provided by the literature descriptions, and all spatiotemporal information ensures objectivity and verifiability.
- Patient information: In the case-cohort, all columns, except for the exposed population (individuals possibly exposed to the pathogen, such as those who shared a meal), describe information about individuals confirmed as patients with *Salmonella* through laboratory diagnosis. Patient symptoms are limited to those commonly emphasized by the U.S. Department of Health & Human Services for *Salmonella* infection, excluding typhoid-specific symptoms such as relative bradycardia, rose spots, hepatosplenomegaly, and others.
- Laboratory test: For the pathogen source, we only consider cases of foodborne illness, classifying contaminated foods based on both the China Food Production Licence Classification Catalogue and the FDA Product Categories and Products. Regarding serovars and serogroups, we adhere to the Antigenic Formulae of the *Salmonella* serovars from the WHO Collaborating Centre for Reference and Research on *Salmonella*, marking all variants, synonyms, and combined serovars with an asterisk (*). Given the rarity of sequence typing data, this dataset only includes MLST typing results, with no recording of wgMLST and cgMLST typing results. The types of antimicrobials and their English abbreviations are referenced in Clinical and Laboratory Standards Institute M100-ED33, which is applicable to clinical management of medication data. We focus on statistically reporting commonly used laboratory diagnostic methods, while separately listing less commonly used methods.
- Clinical management: Treatment medications are exclusively documented for antimicrobial usage and do not include information on supportive therapy drugs. Prognosis is categorized into recovery and death, with cases of adverse outcomes not specifying death not being recorded.

All references and website addresses are available for consultation within the “Sheet3_Reference” repository. The dataset designed for systematic reviews or meta-analyses is separately structured, facilitating the conduct of analyses at different levels of research.

## Technical Validation

Through searches in six major Chinese and English databases, all data were sourced from reputable, publicly available outlets and consisted of peer-reviewed publications. Detailed information about these publications has been incorporated into the dataset. We have established unified and specific standards for search strategy, publication retrieval, screening, and data extraction. To ensure the highest quality of data, authors conducted rigorous triple-checks through manual annotation verification during the data extraction and quality control steps, particularly in the case of meta-analysis materials.

## Usage Notes

The datasets within this archive can be utilized independently to present trends and features of SO. Additionally, they can be integrated with socioeconomic and climate data for correlation analysis or mathematical modeling. The ultimate goal of this study is to utilize a wide range of collected multidimensional parameters for epidemic forecasting, thereby providing relative assurance for human public safety.

**Fig. 3:**
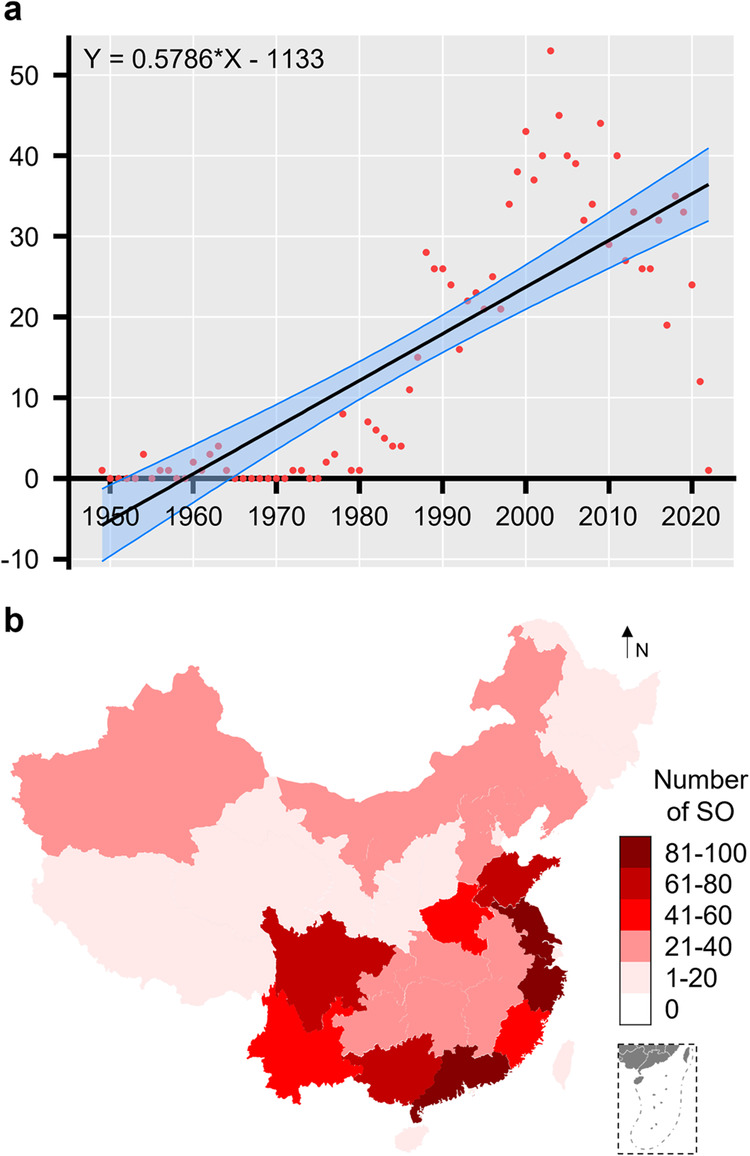
The annual trend and geographical distribution of *Salmonella* outbreaks. (**a**) The variation in the number of *Salmonella* outbreaks with the growth of years is depicted by the black line representing linear regression fit, with the blue semi-transparent area indicating the 95% confidence interval. (**b**) Geographic distribution of *Salmonella* outbreaks in China based on administrative divisions.

Here, we can illustrate the usability of our dataset by focusing on temporal changes and spatial distribution (Fig. 3). By extracting only the columns related to outbreak year and provinces, we can conduct statistical analyses to understand the developmental trends and overall distribution of SO in China. The results show that SO in China is on the rise, with a higher incidence along the southeast coastal regions. The southern areas exhibit a greater SO risk compared to the northern regions. Apart from this, we can conduct more complex analyses and predictions, which will be gradually explored in subsequent stages.

## Data Availability

No code was used in this study.
