# Author Correction: Salmonellosis outbreak archive in China: data collection and assembly

**DOI:** 10.1038/s41597-024-03792-1

**Published:** 2024-08-28

**Authors:** Zining Wang, Chenghu Huang, Yuhao Liu, Jiaqi Chen, Rui Yin, Chenghao Jia, Xiamei Kang, Xiao Zhou, Sihao Liao, Xiuyan Jin, Mengyao Feng, Zhijie Jiang, Yan Song, Haiyang Zhou, Yicheng Yao, Lin Teng, Baikui Wang, Yan Li, Min Yue

**Affiliations:** 1 Department of Veterinary Medicine, Zhejiang University College of Animal Sciences, Hangzhou, 310058 China; 2 Hainan Institute of Zhejiang University, Sanya, 572000 China; 3 Zhejiang Provincial Key Laboratory of Preventive Veterinary Medicine, Hangzhou, 310058 China; 4 State Key Laboratory for Diagnosis and Treatment of Infectious Diseases, National Clinical Research Center for Infectious Diseases, National Medical Center for Infectious Diseases, The First Affiliated Hospital, College of Medicine, Zhejiang University, Hangzhou, 310003 China

**Keywords:** Bacterial infection, Developing world, Bacteriology, Risk factors, Bacterial infection, Developing world, Bacteriology, Risk factors

Correction to: *Scientific Data* 10.1038/s41597-024-03085-7, published online 27 February 2024

In Fig. 1 of this article the wrong version of panel a was used; the original and corrected figures are shown below.

Original Figure 1:
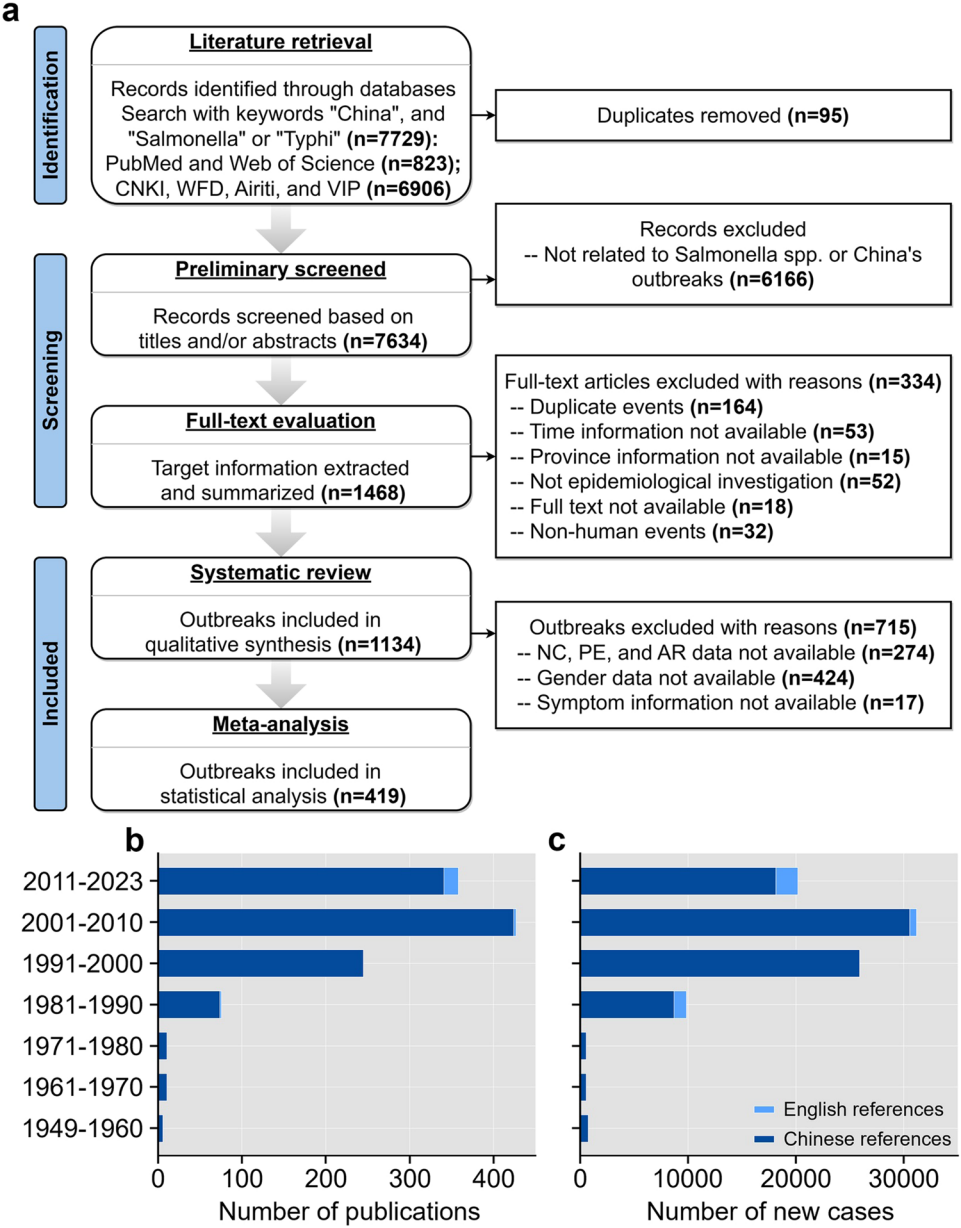


Corrected Figure 1:
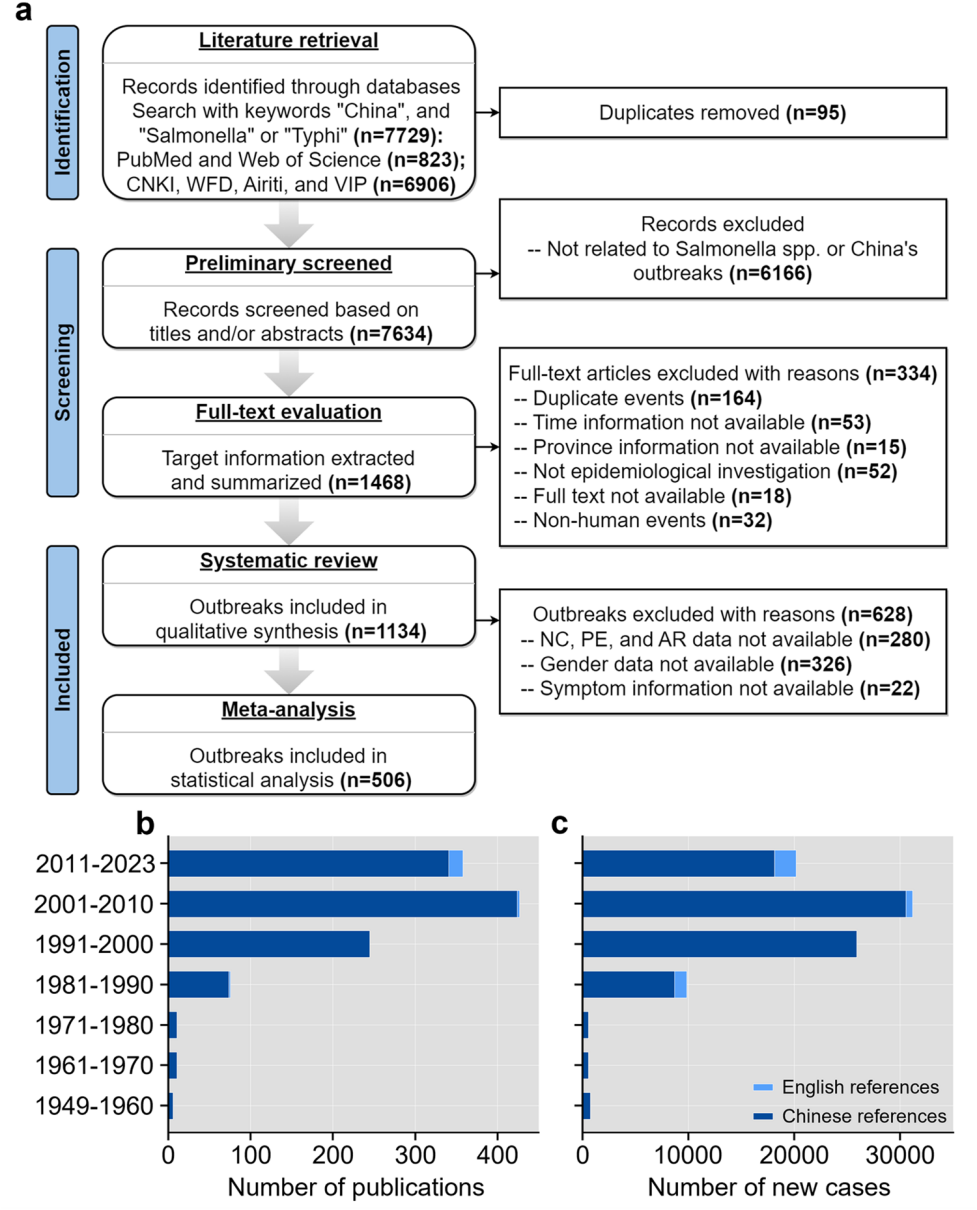


In addition, the figure legends for Fig. 1b and c were inadvertently swapped. The original article has been corrected.

